# Feasibility of resuscitation contrast-enhanced postmortem computed tomography using cardiopulmonary resuscitation technique with chest compression immediately after death

**DOI:** 10.1186/2193-1801-2-663

**Published:** 2013-12-10

**Authors:** Kazunori Iizuka, Namiko Sakamoto, Seiji Shiotani, Atsushi Komatsuzaki

**Affiliations:** Department of Radiology, National Hospital Organization Matsumoto Medical Center, 2-20-30 muraimachiminami, Matsumoto, Nagano, 399-8701 Japan; Department of Forensic Medicine, Hirosaki University Graduate School of Medicine, Hirosaki, Japan; Department of Radiology, Tsukuba Medical Center, Tsukuba, Japan; Department of Radiology, National Hospital Organization Tokyo Medical Center, Meguro, Japan

**Keywords:** Post-mortem computed tomography (PMCT), Contrast-enhanced PMCT (CEPMCT), Cardiopulmonary resuscitation (CPR), Chest compression, CT attenuation value

## Abstract

**Purpose:**

Our purpose was to evaluate image delineation ability of contrast-enhanced post-mortem computed tomography (CEPMCT) using cardiopulmonary resuscitation technique of chest compression, named “resuscitation CEPMCT”.

**Materials and methods:**

Non-traumatically-deceased 15 subjects (7 men; 8 women) aged 19–87 years (mean 61 years) underwent resuscitation CEPMCT. The contrast-enhanced technique, while injecting 100 ml of contrast media from the right cubital vein at a rate of 1 ml/s, chest compression was performed for 2 minutes at a rate of 100 times/min (a total of 200 times). CT attenuation values (Hounsfield Unit: HU) were measured in 8 target vessels: 1) pulmonary artery, 2) coronary artery, 3) ascending aorta, 4) abdominal aorta, 5) celiac trunk, 6) common iliac artery, 7) superior vena cava, and 8) inferior vena cava. One-sided Student’s *t*-test was performed to assess whether measured values were higher than 140 HU by setting p-value at 0.05.

**Results:**

Measured CT values in the 8 vessels were 1) pulmonary artery: 325 ± 140 HU, 2) coronary artery: 240 ± 73 HU, 3) ascending aorta: 321 ± 127 HU, 4) abdominal aorta: 286 ± 96 HU, 5) celiac trunk: 233 ± 62 HU, 6) common iliac artery: 260 ± 114 HU, 7) superior vena cava: 422 ± 187 HU, and 8) inferior vena cava: 301 ± 142 HU, showing significantly higher values than the threshold value of 140 HU. Resuscitation CEPMCT detected one case of pulmonary arterial thromboemboli death.

**Conclusion:**

Resuscitation CEPMCT using chest compression immediately after death has the possibility of detecting thromboembolus in major vessels, despite the simplicity of the technique.

**Electronic supplementary material:**

The online version of this article (doi:10.1186/2193-1801-2-663) contains supplementary material, which is available to authorized users.

## Introduction

Due to the worldwide decline in conventional autopsy rates, the need for and frequency of post-mortem cross-sectional imaging as a complementary, supplementary or alternative method for autopsy have increased worldwide (Brogdon 1998; Swift and Rutty 2006; Oesterhelweg and Thali 2009; Rutty et al. 2012a).

Death cause detection rate of non-traumatic PMCT alone is approximately 30% in general, while PMCT is especially useful in showing fatal hemorrhagic lesions, including cerebral/subarachnoid hemorrhage, aortic dissection, and aortic aneurysmal rupture (Kaneko et al. 2010; Takahashi et al. 2012; Okuda et al. 2013). Thromboembolism of the coronary artery or pulmonary artery is difficult to detect with PMCT; however, contrast-enhanced PMCT (CEPMCT) can detect them (Jackowski et al. 2005; Grabherr et al. 2007; Steffen et al. 2008; Sakamoto et al. 2009; Iizuka et al. 2009; Kikuchi et al. 2010; Ross et al. 2011; Jolibert et al. 2011; Saunders et al. 2011; Roberts et al. 2011; Ross et al. 2012; Rutty et al. 2012b). Several methods of CEPMCT have been developed worldwide. In Western countries, surgical management for embalming is necessary to perform CEPMCT (Morgan et al. 2014). In Japan, CEPMCT is performed by injecting contrast media from the venous route in combination with chest compression. This technique is named “Resuscitation CEPMCT” because the method is similar to that of the cardiopulmonary resuscitation technique (Okuda et al. 2013; Sakamoto et al. 2009; Iizuka et al. 2009; Kikuchi et al. 2010); however, no literature has been published regarding quantitative evaluation of the vascular delineation ability. Herein, we report the image delineation ability of Japanese CEPMCT technique with chest compression, based on retrospectively measured CT attenuation values in major vessels.

## Materials and methods

### Subjects

Our subjects were 15 non-traumatically-deceased patients (7 men and 8 women) aged 19–87 years (mean 61 years) who underwent resuscitation CEPMCT with chest compression between September 2009 and March 2010. Each death was confirmed after subject’s arrival in a state of cardiopulmonary arrest at the emergency room (ER) of National Hospital Organization Tokyo Medical Center. Cardiopulmonary resuscitation (CPR) was performed on all subjects by emergency technicians during transport and in the ER by emergency medical physicians for 30 min. in accordance with the 2010 American Heart Association (AHA) Guidelines for CPR (American Heart Association 2010). CPR included continuous external chest compression, artificial respiration with bag-valve mask ventilation following endotracheal intubation, electric defibrillation, peripheral intravenous catheterization following administration of epinephrine at 1 mg, and infusion. Although the family of each subject did not consent to autopsy, they did consent to PMCT and resuscitation CEPMCT. Causes of death were diagnosed based on a comprehensive evaluation of a subjects’ history of present illness, medical history, laboratory results, and PMCT findings, which included aortic dissection (4 cases), cerebral hemorrhage (3 cases), ischemic heart disease (2 cases), pneumonia (2 cases), gastric cancer (1 case), drug toxicity (1 case), and suffocation (1 case) and pulmonary thromboembolism (1 case).

### Methods

Firstly, PMCT was performed immediately after the confirmation of death using a clinical scanner in the Radiology Department of National Hospital Organization Tokyo Medical Center, with prior approval of the institutional review board. PMCT was performed with an 8-channel multidetector-row CT scanner (Lightspeed Ultra; GE Healthcare, Milwaukee, USA). The imaging parameters for the head, neck, thorax, abdomen, and pelvis were determined for helical scan mode with settings of auto mA (200–400, noise index: 6.0), 120 kV, 0.5 sec/rotation, 1.25 mm collimation, 1.625 pitch, scan speed of 16.75 mm/rotation, and helical thickness of 5 mm.

Secondly, resuscitation CEPMCT was performed as described below. For injecting a contrast media from the right cubital vein, an automatic injector (Dual Shot GX, Nemoto Kyorindo Inc., Japan) was used for the peripheral intravenous catheter retained for infusion during CPR. The contrast media used was iopamidol (Oypalomin 300 Injection Syringe, Konica Minolta Holdings Inc., Japan), which is a non-ionic media generally used in clinical practice. A dose amount of 100 ml was injected at a rate of 1 ml/second. While injecting the contrast media, chest compression was done on the CT table for 2 minutes at a rate of 100 times/minutes (a total of 200 times), in accordance with 2010 AHA Guidelines for CPR (American Heart Association 2010). Scanning parameters for resuscitation CEPMCT were the same as for PMCT.

**Figure 1:**
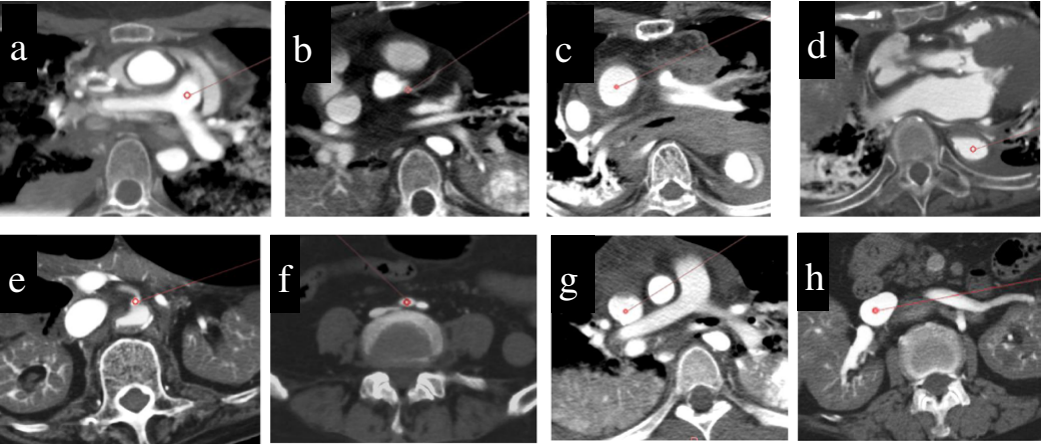
**PMCT of 8 target blood vessels of a 64-year-old man.** A red circle indicates a region of interest in which CT attenuation value (HU) was measured. **a**. Pulmonary artery trunk **b**. Left main coronary artery **c**. Ascending aorta at the level of the tracheal bifurcation **d**. Abdominal aorta at the level of the diaphragm **e**. Root of the celiac trunk **f**. Right common iliac artery immediately after branching from the descending aorta **g**. Superior vena cava at the level of bifurcation pulmonary artery. **h**. Inferior vena cava at the level of renal vein.

**Figure 2:**
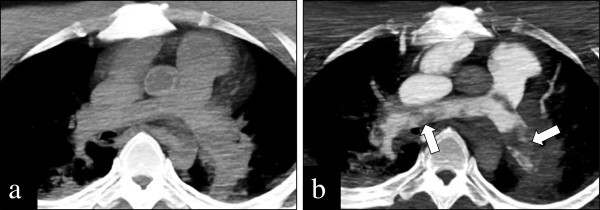
**A 38-year-old man died for thromboembolism of pulmonary artery.** Detection of thrombi is difficult with PMCT **(a)**. On a resuscitation CEPMCT image, CT value of the pulmonary arterial trunk was 269 HU, and that of thromboemboli in the pulmonary artery was 54 HU. The filling defects in the pulmonary artery indicated the presence of thromboemboli **(b)**.

Intra-luminal CT attenuation values of the following 8 vessels were measured: 1) pulmonary artery trunk (Figure 1a); 2) left main coronary artery (Figure 1b); 3) ascending aorta at the level of the tracheal bifurcation (Figure 1c); 4) abdominal aorta at the level of the diaphragm (Figure 1d); 5) root of the celiac trunk (Figure 1e); 6) right common iliac artery immediately after branching from the descending aorta (Figure 1f); 7) superior vena cava at the level of bifurcation pulmonary artery (Figure 1g); and 8) inferior vena cava at the level of renal vein (Figure 1h). A circle-shaped region of interest (ROI) with the diameter of 3 mm was placed in the center of the subject vessels, except for the 2) left main coronary artery and 5) root of the celiac trunk, where the diameter of the ROI was set at 1 mm.

### A threshold value setting for detection of thromboemboli

For detection of thromboemboli, a threshold value of 140 Hounsfield Unit (HU) was chosen as the minimally-necessary enhancement value in the vessel. Generally, CT attenuation value of thrombus is 50–80 HU, plaque (mainly fat and fiber) of the coronary artery is less than 120 HU, and plaque (mainly calcification) of the coronary artery is higher than 120 HU (Schroeder et al. 2004). The contrast discrimination threshold (namely, CT value difference by which radiologists never fails to distinguish among tissues) is approximately 20 HU (Ariji et al. 1993). Therefore, theoretically, thrombus without including calcification can be detected when CT attenuation value in the vessel is higher than 140 HU.

The one-sided *t* test was used to evaluate whether the mean attenuation value of each target vessel was significantly greater than 140HU. A P value of less than 0.05 was considered to be a statistically significant difference.

## Results

Mean CT attenuation values of each targeted vessel are shown in Table 1. All of the 8 examined vessels had CT attenuation values of greater than 140 HU. There was no extravasation of contrast media due to postmortem increased permeability of the vascular wall. In one case having thromboemboli of the pulmonary artery, detection of the thrombi was difficult with PMCT (Figure 2a). On a resuscitation CEPMCT image, CT value of the pulmonary arterial trunk was 269 HU, and that of thromboemboli in the pulmonary artery was 54 HU. The filling defects in the pulmonary artery indicated the presence of thromboemboli (Figure 2b).

**Table 1:** **CT attenuation values measured on contrast-enhanced postmortem CT**

	Case I.D. No.															
	1	2	3	4	5	6	7	8	9	10	11	12	13	14	15	Mean ± SD
1. Pulmonary artery	559	186	234	516	215	341	216	290	371	198	481	234	191	569	269	325 ± 140*
2. Coronary artery	350	265	286	205	198	271	168	226	291	226	210	254	165	388	100	240 ± 73*
3. Ascending aorta	425	310	358	359	221	492	224	317	429	204	335	249	191	593	114	321 ± 127*
4. Abdominal aorta	430	303	346	369	201	303	222	300	355	198	265	258	190	453	97	286 ± 96*
5. Celiac artery	305	241	258	220	189	245	223	289	303	189	220	264	194	287	61	233 ± 62*
6. Common iliac artery	413	235	365	173	182	517	228	280	358	220	231	284	171	187	58	260 ± 114*
7. Superior vena cava	823	236	218	684	241	335	226	447	548	455	628	503	275	420	290	422 ± 187*
8. Inferior vena cava	625	240	214	624	216	279	215	215	333	208	368	230	167	287	298	301 ± 142*

Notes: CT attenuation values are shown as Hounsfield Unit (HU).

*Significantly greater CT attenuation value than a threshold of 140 HU (*p* < 0.05).

## Discussion

The resuscitation CEPMCT technique in the present study using intravenous contrast media injection and chest compression delineated all 8 vessels with CT attenuation values of over 140 HU. A small thrombus might be difficult to detect using enhanced CT, and the negative detection rate of acute pulmonary embolism is reported to be 60% using multidetector CT (Stein et al. 2006). However, CEPMCT is considered capable of detecting relatively large thromboemboli which can be a cause of sudden death. In our study, thrombi were detected in one case of pulmonary arterial thromboemboli death. The formation of postmortem blood clots is closely linked to the length of the agonal interval (Ross et al. 2012). In the case of a sudden death, plasminogen activator is released into the vessel, resulting in increased fluidity of blood (Shiotani et al. 2002). Therefore, PMCT performed immediately after death can differentiate thromboemboli from postmortem coagulation. This infers the feasibility of resuscitation CEPMCT for detection of thromboemboli at least in the pulmonary arteries, which has been difficult to do with PMCT alone.

Chest compression during CPR increases blood pressure, and generates cardiac output of approximately one-third to one-fourth of the normal state of a living body (Jackson and Freeman 1983; Paradis et al. 1989). This phenomenon enables delineation of our resuscitation CEPMCT with chest compression. When chest compression is not applied for CEPMCT, injected contrast media migrates from the right atrium to the inferior vena cava, without flowing into the right chamber or left heart (Morgan et al. 2014). With chest compression, contrast media injected from the upper arm (cubital vein) enters the right atrium, moves into the right ventricle, pulmonary artery, pulmonary vein, left atrium, left ventricle, aorta, with the enhanced image of the arterial route being delineated.

There are two drawbacks to this study. One is that all of the subjects were non-traumatically deceased patients. In traumatic cases where chest compression is not effective, such as multiple rib fracture and significant loss of blood volume from bleeding, contrast-enhancement would be insufficient due to the lack of effective perfusion of the contrast media in the body. Another drawback is that it is uncertain whether the injected amounts, concentrations, and flow rate of the contrast media and the time of chest compression we performed were optimal for resuscitation CEPMCT.

We surmise that increased intravascular CT values would be obtained with increased amounts and concentrations of contrast media. On the other hand, an excessive number of chest compressions will widely diffuse contrast media in the body, which may obscure intra-vascular CT values. The optimum imaging conditions should be further investigated.

In conclusion, resuscitation CEPMCT with the combination of contrast media injection and chest compression immediately after death is believed to be a relatively simple additional technique to PMCT for the detection of vascular disease-related causes of death.

## Availability and requirements

-  **Project name:** none
-  **Project home page:** none
-  **Operating system(s):** none
-  **Programming language:** none
-  **Other requirements:** none
-  **License**: none
-  **Any restrictions to use by non-academics:** none

## Authors’ original submitted files for images

Below are the links to the authors’ original submitted files for images. Authors’ original file for figure 1 Authors’ original file for figure 2
