# Supplementary figures and images for: Feasibility of resuscitation contrast-enhanced postmortem computed tomography using cardiopulmonary resuscitation technique with chest compression immediately after death
Source: Springerplus. 2013 Dec 10;2(1):663. doi: 10.1186/2193-1801-2-663 (PMC3866376; doi:10.1186/2193-1801-2-663)

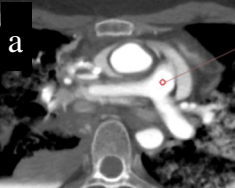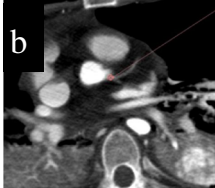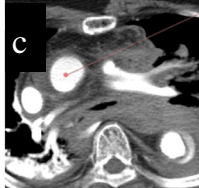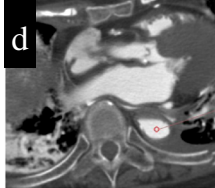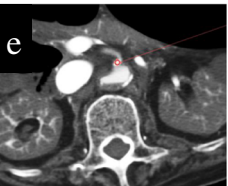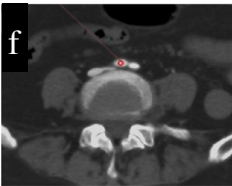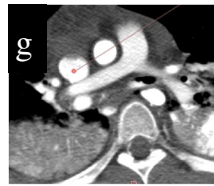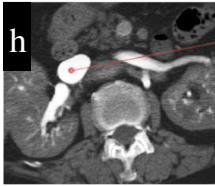

Supplement: Supplementary file 1 — Authors’ original file for figure 1 [file 40064_2013_730_MOESM1_ESM.pdf]

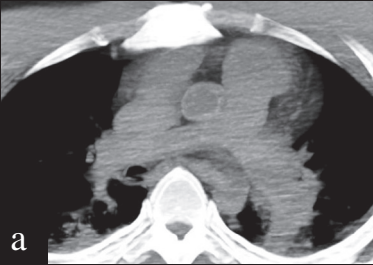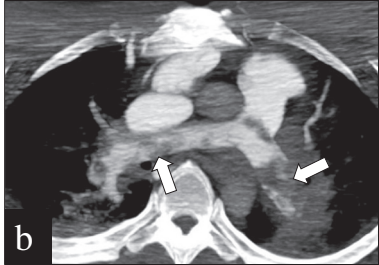

Supplement: Supplementary file 2 — Authors’ original file for figure 2 [file 40064_2013_730_MOESM2_ESM.pdf]
